# The KIzSS network, a sentinel surveillance system for infectious diseases in day care centers: study protocol

**DOI:** 10.1186/1471-2334-12-259

**Published:** 2012-10-15

**Authors:** Remko Enserink, Harold Noel, Ingrid HM Friesema, Carolien M de Jager, Anna MD Kooistra-Smid, Laetitia M Kortbeek, Erwin Duizer, Marianne AB van der Sande, Henriette A Smit, Wilfrid van Pelt

**Affiliations:** 1 Center for Infectious Disease Control (Epidemiology and Surveillance Unit), National Institute for Public Health and the Environment (RIVM), Bilthoven, The Netherlands; 2 Center for Infectious Disease Control (Laboratory for Infectious Diseases and Perinatal Screening), National Institute for Public Health and the Environment (RIVM), Bilthoven, The Netherlands; 3 Julius Center for Health Sciences and Primary Care, University Medical Center Utrecht, Utrecht, The Netherlands; 4 Laboratory for Infectious Diseases, Department of Research and Development, Groningen, The Netherlands

**Keywords:** Day care, Infectious disease, Public health, Surveillance, Study design

## Abstract

**Background:**

Day care-associated infectious diseases are widely recognized as a public health problem but rarely studied. Insights into their dynamics and their association with the day care setting are important for effective decision making in management of infectious disease control. This paper describes the purpose, design and potential of our national multi-center, day care-based sentinel surveillance network for infectious diseases (the KIzSS network). The aim of the KIzSS network is to acquire a long-term insight into the syndromic and microbiological aspects of day care-related infectious diseases and associated disease burden and to model these aspects with day care setting characteristics.

**Methods/design:**

The KIzSS network applies a prospective cohort design, following day care centers rather than individual children or staff members over time. Data on infectious disease symptoms and related morbidity (children and staff), medical consumption, absenteeism and circulating enteric pathogens (children) are collected on a daily, weekly or monthly basis. Every two years, a survey is performed to assess the characteristics of participating day care centers.

**Discussion:**

The KIzSS network offers a unique potential to study infectious disease dynamics in the day care setting over a sustained period of time. The created (bio)databases will help us to assess day care-related disease burden of infectious diseases among attending children and staff and their relation with the day care setting. This will support the much needed development of evidence-based and pragmatic guidelines for infectious disease control in day care centers.

## Background

Children cared for in day care centers (DCCs) are at increased risk of acquiring respiratory and gastrointestinal disease compared to children cared for at home [1-5]. Clearly, the DCC environment provides a setting conducive to increased transmission of infectious disease. DCCs often represent crowded facilities that provide care for an immunological immature population of children that have little notion of basic hygiene. In addition, interventions for infectious disease control are often not available (vaccination), socially undesirable (exclusion) or not effective (cohorting) as small children can be infectious before and after becoming symptomatic [6]. Finally, the infectious disease burden not only concerns the attending child. Day care-associated infectious disease transmission has been considered a public health problem since 1984 [7]. Infectious pathogens, including their antiviral or antimicrobial resistance properties, may readily transmit via children, caretakers, parents and families [8] into the society at large, resulting in additional infectious disease burden [9], health care utilization [10] and work absenteeism [11].

The potential impact of day care-related infectious diseases on both the child attending day care, as well as the society in general, is substantial. It is therefore pivotal that DCCs are supported in their efforts to control and prevent infectious disease transmission within their facility. However, current research initiatives are often not intended nor able to provide that support. Notifiable infectious disease registries of Municipal Health Services, in addition to suffering from substantial underreporting as DCCs are often unaware of reporting requirements and primarily focus on infectious disease outbreaks, neglecting sporadic infectious disease events. Furthermore, studies concerning infectious disease dynamics in day care centers often consider the DCC as one risk factor rather than a complex setting in which many factors may influence infectious disease occurrence [12-14].

As these registries and research initiatives provide DCCs with only a limited insight into the infectious disease dynamics at their facilities, they rarely succeed in guiding DCCs in determining the most effective and pragmatic interventions for infectious disease control management. DCCs need a better understanding of the infectious disease dynamics of their child – and staff – population, as well as its preventable fraction. DCCs contributing to a surveillance network for infectious diseases may judge this directly by benchmarking their findings with that of the other DCCs.

National surveillance systems for monitoring of infectious diseases have been implemented before in nursing homes [15], hospitals [16], general practices [17] and schools [18]. To our knowledge however, the concept has not yet been applied in day care centers. There are currently approximately 6000 DCCs in the Netherlands, providing care for half of the approximate 0.7 million Dutch population of children aged between 0 and 4 years [19,20]. Since 2010, the Dutch National Institute for Public Health and the Environment (RIVM) therefore maintains a day care based sentinel surveillance network for infectious diseases throughout the Netherlands; Dutch acronym: KIzSS. The aim of the KIzSS network is to acquire a long-term insight into the syndromic and microbiological aspects of day care-related infectious diseases and to model these aspects with the DCC setting. The present paper describes the purpose, design and potential of the KIzSS network.

## Methods/design

### Study design and setting

The KIzSS network has a prospective cohort design, following day care centers rather than individual children or staff members over time. Data on infectious disease symptoms and related morbidity (children and staff), medical consumption and absenteeism, circulating enteric pathogens (children) and day care center characteristics, are collected on structural bases. The general organization and logistics regarding the data collection process was piloted during and evaluated at the end of the first surveillance year. This evaluation provided useful feedback that, with some minor logistical adjustments, data collection was feasible in participating centers. The KIzSS network is operated by a research team that includes (medical) microbiologists, epidemiologists, a research assistant and laboratory technicians.

### Ethical approval

This study is conducted according to the principles of the Declaration of Helsinki. The Dutch Central Committee on Research involving Human Subjects in Utrecht, The Netherlands, gave ethical approval to conduct this study. No subject-identifiable results are generated. The design was developed to interfere as little as possible with the wellbeing of the children. Parents or guardians of children attending participating DCCs were informed by letter of the purpose and design of the study. An information form was attached that parents could return if they did not want to let their child participate in the study.

### Recruitment of DCCs

Initial recruitment amongst Dutch DCCs took place from November 2009 to April 2010 using a continuously updated database provided by the ministry of Education, Culture and Sciences (N=3913 DCCs, creation date: January 2007). From March 2012 onwards, yearly recruitment took place using a continuously updated database operated by the Ministry of Social Affairs and Employment (6000 DCCs, creation date: January 2011). As parents receive child care subsidy only if their child attends a day care center that is registered in this database, it is assumed that all DCCs in the Netherlands are approached.

**Figure 1:**
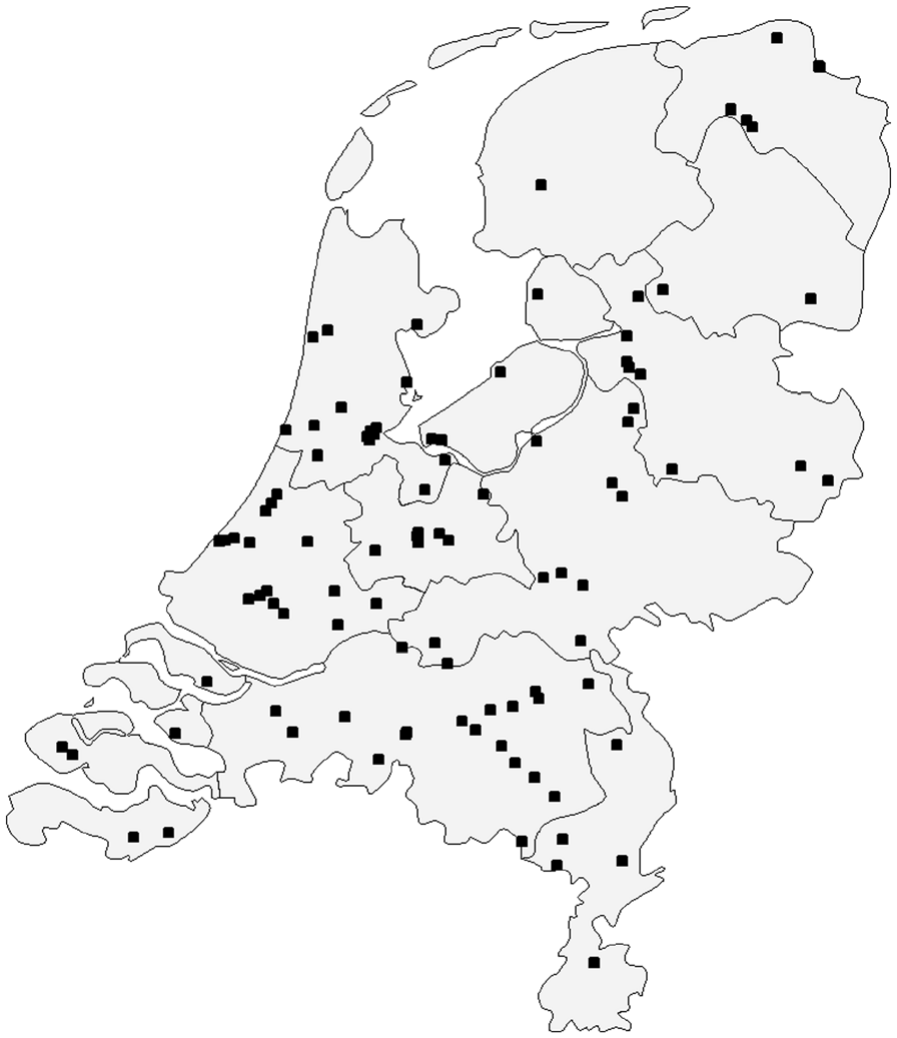
Geographical location of DCCs currently participating in the KIzSS network in the Netherlands (March 2012).

During all recruiment periods, DCCs are eligible to participate in surveillance activities if they provide full time day care for children less than 4 years of age and have an internet connection. Assuming that the number of DCCs interested in participation will be low given the workload involved, no further selection criteria are used. Included DCCs are either instructed via email and telephone or visited by the research team if needed. During these instructions, aims, setup and workload of the study are discussed. DCCs are asked to participate for at least one year. In return, the DCCs receive minor incentives throughout the study year, including a report (see “Dissemination of results” for more details), a small financial donation and one or more site visits (additional to the inclusion visit) if requested. An overview of the DCCs currently participating in the KIzSS network (N=104) is presented in Figure 1.

### Data collection

An overview of the KIzSS network design and data collection parameters is presented in Table 1 and is further explained in the following sections.

**Table 1:** Design of the KIzSS sentinel surveillance network

**Survey day care settings**		**Syndrome surveillance**		**Microbiological surveillance**	
**Numerator Denominator Data**	-	**Numerator Denominator Data**	Number of syndromic episodes Child/staff-weeks at risk	**Numerator Denominator Data**	Number of positive samples Number of fecal samples
*Demographics*	Children attending, capacity, group structure, child/caregiver ratio, s, degree of urbanization, religious background etc.	*Demographics*	Date of birth, gender, co-morbidity	*Demographics*	Date of birth, gender, date of sampling
		*Symptoms*	Fever, ear ache, runny ear, diarrhea, vomiting, coughing, exanthema, other symptoms	*Bacteria*	Escherichia, Salmonella, Shigella, Campylobacter, Clostridium
*Facility design*	Presence of sandpit, paddling pool, toilet, sink, nappy changing area, paper towels, animals etc.	*Syndromes*	Chicken pox, impetigo, common cold, eye infection, otitis media, gastroenteritis, influenza-like illness, other syndromes	*Viruses*	Norovirus, Sapovirus, Rotavirus, Adenovirus, Astrovirus
*Hygiene policy*	Hand/food hygiene, group/staff mixing, hand washing/cleaning policies, covering sandpits/pools etc.	*Morbidity*	Duration of illness, absenteeism, exclusion, medical consultation, hospital admission, medication	*Parasites*	Giardia, Cryptosporidium, Dientamoeba
*Surveillance and control*	Documentation of illness, co-morbidity, medication use, cohorting/exclusion, public health notification etc.			*Morbidity*	Diarrhea, vomiting
		*Population at risk*	Number of attending children Number of attending staff	*Antimicrobial resistance*	Standard panel of 14 antibiotics and ESBL producing bacteria
**Reporting**	Per DCC By DCC manager Using questionnaire Via mail Every two years	**Reporting**	Per child/staff member By DCC staff member Using paper and/or digital logbook Via internet Daily (numerator)/weekly (denominator)	**Reporting**	Per child By DCC staff member Using feces envelope Via mail Every month

#### Survey

During recruitment periods every Dutch DCC is asked to fill in a questionnaire addressing baseline DCC setting characteristics. These include questions regarding the sociodemographics of the DCC (number of children attending, capacity, group structure arrangements, child/staff ratio, socioeconomic status, degree of urbanization, religious background, etc.), facility design (presence of sandpit, paddling pool, toilets/sinks, nappy change area, paper towels, presence of animals, etc.), DCC hygiene policies (hand/food hygiene, group/staff mixing, cleaning frequency of toilets/kitchen/toys/beds/sandpits, etc.) and DCC policies for surveillance and control of infectious disease (documentation of illness episodes, allergies and chronic conditions, medication use, exclusion and cohorting policies, public health services notification, etc.).

#### Syndrome surveillance

Participating DCCs are asked to anonymously report each child or staff member experiencing predefined illness symptoms (fever, ear ache, runny ear, diarrhea, vomiting, coughing, exanthema) and/or syndromes (chickenpox, impetigo, common cold, and eye infection) on a daily basis from Monday - Sunday. If a child experiences symptoms or syndrome other than prespecified, DCCs can specify these in a free text field. Predefined symptoms/syndromes and additional clinical information are used to postdefine additional syndrome definitions (gastroenteritis, influenza-like illness and otitis media). Both predefined and postdefined symptoms/syndromes are based on simplicity of syndrome presentations and comparability with current national day care guidelines for infectious diseases. An overview of the symptom and syndrome definitions is presented in Table 2.

**Table 2:** Predefined and postdefined symptoms and syndromes of infectious disease

**Predefined symptoms**		**Predefined syndromes**		**Postdefined syndromes**	
**Symptom**	**Definition**	**Symptom**	**Definition**	**Syndrome**	**Definition**
Fever	Sudden onset of fever (≥ 38°C) and/or warm to the touch with suspicion of fever with or without other symptoms	Chickenpox	Sudden rash of small red bumps, followed by itchy blisters and crust	Gastroenteritis	Diarrhea and/or vomiting
Ear ache	Sudden onset of aching ear, with or without listlessness and ear infection confirmed by physician or parent(s)	Impetigo	Expanding small red papules followed by a honey-colored crust.	Influenza-like illness	Fever with ≥ 1 general symptoms (headache and/or febrile feeling and/or listlessness) and ≥ 1 respiratory symptoms (coughing and/or runny nose and/or throat ache)
Runny ear	Light yellow, watery purulent discharge from ear that may or may not smell	Common cold	Sudden onset of continuous sneezing and/or coughing and/or headache and/or throat ache and/or coughing with or without fever (≥ 38°C)	Otitis media	Ear ache and/or runny ear with fever and/or common cold
Diarrhea	Sudden, non-chronic, onset of > 3 episodes of watery stools per day	Eye infection	Red eyes and swollen eyelids with or without yellow/green mucal discharge from eye		
Vomiting	Sudden, non-chronic, onset of > 3 emetic episodes per day				
Coughing	Sudden and frequent occurring tussis				
Exanthema	Spots on skin, rash				
Other symptoms	-				

Each child or staff member may experience one syndromic episode per reporting. Per reporting additional data are collected on age, gender, allergy-related conditions as well as possible absenteeism, exclusion, medical consultation, hospital admission and use of antiviral/antibiotic medication.

All children and staff experiencing an illness episode at the DCC or those that (are) call(ed) in sick should be reported. An ill child or staff member has to be fully recovered for 7 days before being reported ill again. DCCs are contacted regularly by telephone to ensure maximal surveillance response. Screening of illness episodes amongst DCC attending children and staff is performed by all DCC personnel, but only one qualified person per DCC records these episodes on a daily basis using a centralized paper logbook. This logbook is submitted digitally using the web-based registration tool OSIRIS [21]. Finally, DCCs are asked to report the number of attending children at the DCC per week on a weekly basis as this will fluctuate due to e.g. holiday seasons. This defines the child population at risk per week. A researcher checks all reports for completeness on a monthly basis. Non-completed or unclear reports are sent back to the DCC with digital comments. The DCC gets the opportunity to make appropriate changes or comments and resend the report.

#### Microbiological surveillance

20 DCCs participating in the network are asked to randomly take a fecal sample from 10 individual children on a monthly basis in addition to their syndrome surveillance activities. Sampling is performed during the first or last two weeks of every month (2 groups) and occurs regardless of the child's infectious disease status. For each sample, child demographics (age, gender) and, if any, digestive symptoms (vomiting, diarrhea) are collected. Unpreserved fecal samples are stored at the DCC at 4°C upon sending to the laboratory for molecular detection of bacterial, viral and parasitic gastrointestinal pathogens.

#### Molecular detection of bacterial gastrointestinal pathogens

Molecular detection of bacterial gastrointestinal pathogens, including *Salmonella enterica, Campylobacter jejuni, Clostridium difficile, Yersinia enterocolitica, Shigella spp*., shigatoxin producing *E. coli* (STEC), enteroaggregative *E. coli* (EAEC), and typical and atypical enteropathogenic *E. coli* (EPEC), is performed using four internally controlled quantitative real-time multiplex polymerase chain reactions (qPCRs) as described previously [22]. Fecal materials, including fecal suspensions and total nucleic acid isolates, are sent to other laboratories for further molecular typing of viral and parasitic gastrointestinal pathogens (RIVM laboratory) and antimicrobial resistance (Central Veterinary Institute laboratory, CVI). Finally, all remaining materials are stored at −80°C in a central biobank for future reference.

#### Molecular detection of viral gastrointestinal pathogens

Molecular detection of viral gastrointestinal pathogens, including norovirus, adenovirus, sapoviruses, astrovirus and rotavirus is performed using a random priming step followed by multiple internally controlled multiplex PCR assays as described in one of our previous studies [23]. Genotyping of the different viruses is performed by partial genome sequencing of the capsid gene (norovirus, adenovirus, sapoviruses, astrovirus), or using PCR-based genotyping protocols (rotavirus) as described previously.

#### Molecular detection of parasitic gastrointestinal pathogens

Molecular detection of parasitic gastrointestinal pathogens, including *Giardia lamblia*, *Cryptosporidium sp* and *Dientamoeba fragilis*, is performed using one internally controlled qPCR as described previously [24].

#### Detection of antimicrobial resistance, including ESBL

A random subset of approximately 40 unpreserved fecal samples, in which all DCCs collecting feces are represented, are selected every month for detection of a panel of 14 antimicrobial resistance markers using *E.coli* producing Extended Spectrum Béta-Lactamases (ESBL) as indicator-bacterium as described previously [25].

### Statistical analyses

Weekly syndrome incidence is estimated as the number of children or staff with (a) specific symptom(s)/syndrome(s) divided by the total number of child or staff weeks at risk. As children do not attend day care every day of the week, we will model a syndrome dependent correction factor to adjust for syndrome episodes missed due to children getting ill and recovering in between DCC attendance days. Monthly pathogen prevalence is estimated as the number of fecal samples positive for a specific pathogen divided by the total number of fecal samples analyzed that month respectively. Associations between the time-series of syndrome incidence and pathogen prevalence within the DCC population and the general population will be explored using standard regression models as described previously [26]. Associations between the occurrence of syndromes and pathogen trends and DCC characteristics will be analyzed, amongst others, using Poisson multiple regression techniques. Incidence figures will be adjusted for differences between age categories, socioeconomic status (SES) and degree of urbanization in addition to autocorrelation issues. Data are analyzed using the statistical software package STATA/SE 11.2 for Windows.

### (Cost-) Effectiveness and representativeness

The syndromic and microbiologic surveillance activities will be performed for at least 4 and 3 years respectively. Continuation of both, thereafter, will depend on evaluation of their cost-effectiveness, additional research questions posed and future political decisions made concerning DCC quality assurance. The operating costs for the syndromic surveillance component mainly concern hiring a research assistant and PhD student in addition to the costs made for designing/distributing software and relevant documentation. The operating costs for the microbiological surveillance component mainly include laboratory expenditures for performing relevant microbiological analyses.

Estimating the network’s effectiveness will include assessing its performance and representativeness relative to its added scientific value. The performance will be defined as the number of weeks DCCs reported denominator information for syndrome surveillance divided by the total number of weeks these DCCs participated in the network. The representativeness of the DCC cohort will be assessed by comparing DCC characteristics such as socioeconomic classification, degree of urbanization, facility design, and hygiene practices between cohort participants and nonparticipants. In addition, a principal component analysis will be performed to assess whether the overall variation in combinations of DCC characteristics in participants reflects the overall variation among all DCCs.

### Dissemination of results

Self-reported incidence estimates of respiratory, diarrhoeal and exanthematous episodes and, if appropriate, prevalence estimates of circulating gastrointestinal pathogens are communicated to the DCCs in annual reports. These reports allow DCCs to compare their facility anonymously to those of other DCCs and national aggregated figures. The role of DCC hygiene practices on DCC related- infectious disease occurrence will be communicated to the DCCs via the Dutch Health Authorities and the scientific community via publications and conferences.

## Discussion

This article describes the purpose, design and potential of a national sentinel surveillance system in child day care for infectious diseases, including syndromes, related morbidity, circulation of gastrointestinal pathogens and risk factors thereof with respect to the DCC setting.

The major strength of the KIzSS network is the almost real-time, long-term syndrome and microbiological surveillance at the level of the DCC setting. This information offers great potential for understanding infectious disease dynamics in day care. Syndrome surveillance allows for the quantification of the incidence and infectious disease burden of most common diarrheal, respiratory and exanthematous infections (both sporadic and clustered) over time. Microbiological surveillance will provide valuable information on the dynamics of circulation of gastrointestinal pathogens, including antibiotic resistance patterns, over time. In addition, the biobank generated due to storage of remaining fecal material can be used for future reference to study e.g. yet unknown circulating microorganisms in day care with pathogenic potential. Combining syndrome and microbiological surveillance will generate historical baseline data for benchmarking DCCs and might facilitate rapid and comprehensive assessment of future impact of (shifts in) seasonal syndrome and/or microbiological trends of infectious diseases. By studying the influence of the DCC setting herein, the KIzSS study will aid in highlighting the most efficient and pragmatic infectious disease control practices for day care professionals. In addition, the network might be used to monitor the effectiveness of future day care targeted intervention strategies. For example, by comparing disease burden in day care in pre- and post intervention eras. Finally, the KIzSS network could be linked to previous regional or national general population, GP and hospital studies or surveillance registries, allowing us to study whether infectious disease trends in DCCs reflect or perhaps drive infectious disease patterns in the general community.

Some possible limitations also need to be addressed. A consequence of the network’s ecological design is that no subject identifiable results are generated, making it difficult to study pathogen-disease interactions at the individual level. This could lead to ecological fallacies in which correlations found at the DCC level are assumed to apply at the individual level as well, which may not be the case. However, this does not concern the KIzSS network since it focuses on the DCC setting rather than on the individual attending children. Furthermore, as reporting of our surveillance parameters is voluntary, incidences of infectious disease and related burden might be underestimated. On the other hand, DCC attending children do not have to seek out medical care first before being included in our study as is the case in many other studies. This may in turn lead to more realistic incidence estimates compared to general practitioner or hospital-based studies that only receive the more serious cases. Finally, due to budgetary constraints, microbiological surveillance is limited to pathogens found in feces.

A major challenge for the future will be to provide the KIzSS network with a stable, sustainable foundation and a dedicated, robust management. After having proved its sustainability, ease-of-use and value to the DCC and research community alike, the KIzSS network might be easily adapted to incorporate additional syndrome groups, health-related events and (respiratory) pathogens of minor and major concern, further expanding the potential of its (bio)databases for studying infectious disease dynamics in day care centers.

## Abbreviations

CVI: Central Veterinary Institute laboratory; DCC: Day Care Center; EAEC: Enteroaggregative E. Coli; EPEC: Typical and Atypical Enteropathogenic E. Coli; ESBL: Extended Spectrum Béta-Lactamases; KIzSS: Kinderdagverblijven Infectieziekten Surveillance Systeem; RIVM: Dutch National Institute for Public Health and the Environment; STEC: Shigatoxin Producing E. Coli; qPCR: quantitative Real-time Multiplex Polymerase Chain Reaction.

## Competing interests

This study has been internally funded and has not received any funding nor assistance from a commercial organization. The authors declare that they have no competing interests.

## Authors’ contributions

All authors have equally contributed to the design of the study and manuscript preparation. All authors have read and approved the final manuscript.

## Funding sources and related paper presentations

Ministry of Health.

## Pre-publication history

The pre-publication history for this paper can be accessed here:

http://www.biomedcentral.com/1471-2334/12/259/prepub
